# Ursolic Acid Attenuates Atherosclerosis in ApoE^−/−^ Mice: Role of LOX-1 Mediated by ROS/NF-κB Pathway

**DOI:** 10.3390/molecules23051101

**Published:** 2018-05-07

**Authors:** Qiu Li, Wenwen Zhao, Xi Zeng, Zhihui Hao

**Affiliations:** 1 State Key Laboratory of Quality Research in Chinese Medicine, Institute of Chinese Medical Sciences, University of Macau, Macau SAR 999078, China; liqiu370725@126.com; 2 Department of Pharmacology, College of basic Medicine, Qingdao University, 308 Ningxia Road, Qingdao 266000, China; wenwenzhao0313@163.com (W.Z.); hf9079@163.com (X.Z.); 3 Agricultural Bio-Pharmaceutical Laboratory, Qingdao Agricultural University, Qingdao 266000, China

**Keywords:** atherosclerosis, ROS, HUVECs, LOX-1

## Abstract

Atherosclerosis, a chronic inflammatory disease, is a major contributor to cardiovascular diseases. Ursolic acid (UA) is a phytonutrient with widely biological effects including anti-oxidative, anti-inflammatory, and so on. At present, the effect of UA on atherosclerosis and the mechanism of action are still obscure. This study focused on investigating the effects of UA on atherosclerosis both in vivo and in vitro. We first selected LOX-1 as our target, which was reckoned as a new promising receptor for treating atherosclerosis. The evaluation in vitro suggested that UA significantly decreased endothelial LOX-1 expression induced by LPS both in mRNA and protein levels. Pre-treatment of UA also inhibited TLR4/MyD88 signaling activated by LPS. Moreover, UA reduced ROS production and suppressed the activation of NF-κB stimulated by LPS. Particularly, the evaluation in vivo further verified the conclusion obtained in vitro. In ApoE^−/−^ mice fed with an atherogenic diet, both UA (100 mg/kg/day) and simvastatin significantly attenuated atherosclerotic plaque formation and shrunk necrotic core areas. The enhanced expression of LOX-1 in atherosclerotic aorta was also dramatically decreased by administration of UA. Taken together, these results suggested that UA, with anti-atherosclerotic activity through inhibition of LOX-1 mediated by ROS/NF-κB signaling pathways, may become a valuable vascular protective candidate for the treatment of atherosclerosis.

## 1. Introduction

Atherosclerosis, a chronic inflammatory disease of the arterial wall, is a major cause of morbidity and mortality worldwide and is marked by the formation of atherosclerotic plaques [1]. One critical event in the initiation of atherosclerotic plaques is the uptake of ox-LDL [2]. LDL uptake is principally mediated by a variety of specific receptors including SR-A I/II, CD36, SR-BI, etc. [3]. Recently, LOX-1 has been identified as the main endothelial receptor for ox-LDL [4,5]. LOX-1 is a 50 KD transmembrane protein highly expressed on macrophages, vascular smooth muscle cells, and, especially, endothelial cells. It plays vital roles in the pathogenesis of atherosclerosis [6]. Disturbance of LOX-1-mediated signaling pathways has been proposed as a potential strategy for anti-atherosclerotic drug discovery.

TLR4-MyD88 pathway leads to activation of the transcription factor NF-κB, thereby influencing inflammatory responses. Oxidative stress is characterized by an increased production of free oxygen radicals [7]. Numerous clinical studies have demonstrated that oxidant stress is closely related to different risk factors of atherosclerosis such as hypercholesterolemia [8], hypertension [9], diabetes [10], and smoking [11]. Oxidative stress can promote conversion of LDL to atherogenic ox-LDL, contributing to the atherosclerotic plaque formation [12]. Besides, oxidant stress is closely correlated with endothelial dysfunction [13] and promotes vascular inflammatory response [14]. Furthermore, TLRs-NF-κB pathway has been reported to participate in the anti-atherosclerotic effect of several natural products such as quercetin, pycnogenol, and procyanidins [15,16,17].

Ursolic acid (UA) is a pentacyclic triterpenoid found in many herbs and spices like rosemary and thyme, especially in valuable Chinese medicinal herbs such as *Fructus Ligustrum lucidum* [18] and *Forsythiae fructus* [19]. It shows potentially beneficial activities in treating cardiovascular disease due to its anti-oxidative [14], anti-inflammatory effects [20], and other biological activities [21,22]. However, it also demonstrates potential adverse effects. Messner et al. [1] reported the pro-atherogenic effects of UA. There is a controversy around the effect of UA on atherosclerosis. This study is to investigate the effect of UA on cells in vitro; high-fat, diet-induced ApoE^−/−^ mice in vivo; and related mechanisms such as LOX-1 expression in endothelial cells. Our findings may supply a new sight for illustrating role of UA, which benefits the development of the anti-athrogenic drug.

## 2. Results

### 2.1. UA Decreased LPS-Induced LOX-1 Expression in HUVECs

**Figure 1:**
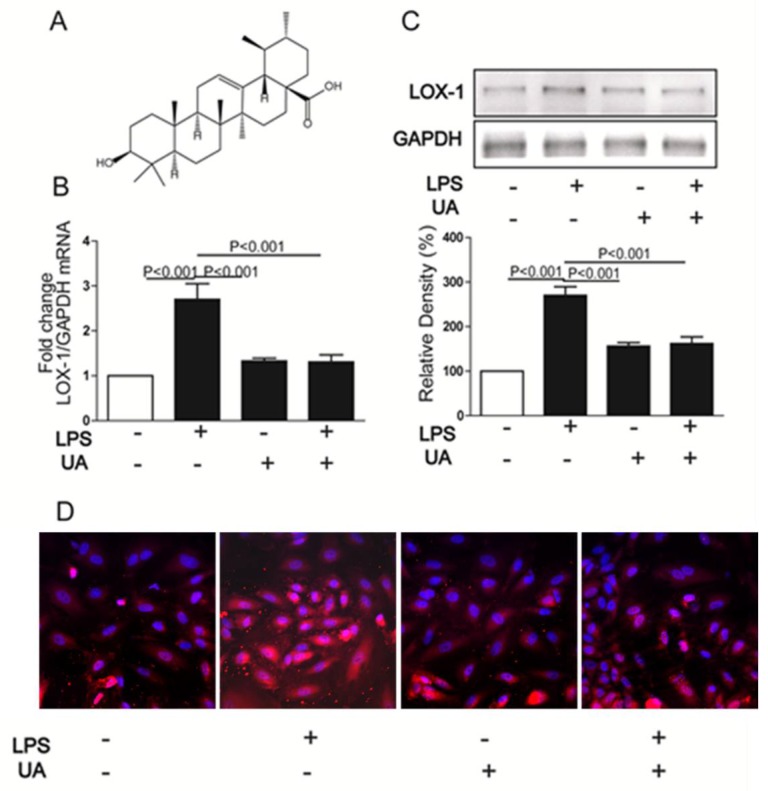
The structure of UA (**A**); Cells were pretreated with UA (1 μM) for 1 h and then stimulated with LPS (5 mg/mL) for 24 h; LOX-1 mRNA (**B**), protein (**C**), and localization on the membranes (**D**) were detected by real-time PCR, western blotting, and immunofluorescence (60×), respectively. UA, ursolic acid.

Firstly, the cytotoxic effect of UA on HUVECs was determined by MTT assay. Results showed that UA was cytotoxic to HUVECs at 50 μM (Figure S1). To minimize the cytotoxic effect of UA on cell viability, 1 μM UA was chosen for further study. Compared with untreated HUVECs, 24 h stimulation with LPS increased LOX-1 expression at both mRNA and protein levels, which were dramatically inhibited by UA pretreatment (Figure 1B,C). Furthermore, immunofluorescence results showed that UA blocked LPS-induced LOX-1 expression localizing on the cell membrane (Figure 1D).

### 2.2. UA Inhibited LPS-Induced LOX-1 Expression via TLR4/MyD88 Pathway

**Figure 2:**
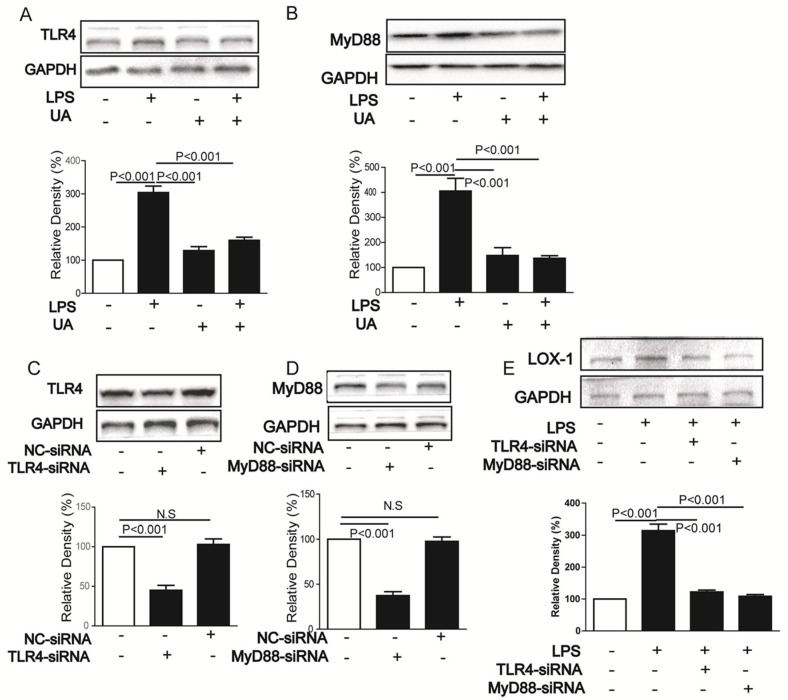
Cells were pretreated with UA (1 μM) for 1 h and then stimulated by LPS (5 mg/mL) for 24 h; expressions of TLR4 and MyD88 were determined by western blotting (**A**,**B**). Cells were transfected with siRNAs for TLR4 (**C**) and MyD88 (**D**) and then stimulated with LPS (5 mg/mL) for 24 h and the LOX-1 expression were determined by western blotting (**E**). Cont, control; NC-siRNA, negative control siRNA. UA, ursolic acid; N.S, no significant differences.

TLR4/MyD88 signal pathway is involved in LPS-induced inflammation [23]. As expected, UA reversed LPS-induced TLR4 and MyD88 protein expressions (Figure 2A,B). Furthermore, silence of either TLR4 or MyD88 significantly decreased LOX-1 expression (Figure 2C,E).

### 2.3. UA Reduced ROS Generation and Decreased NF-κB Activity to Block LOX-1 Expression

**Figure 3:**
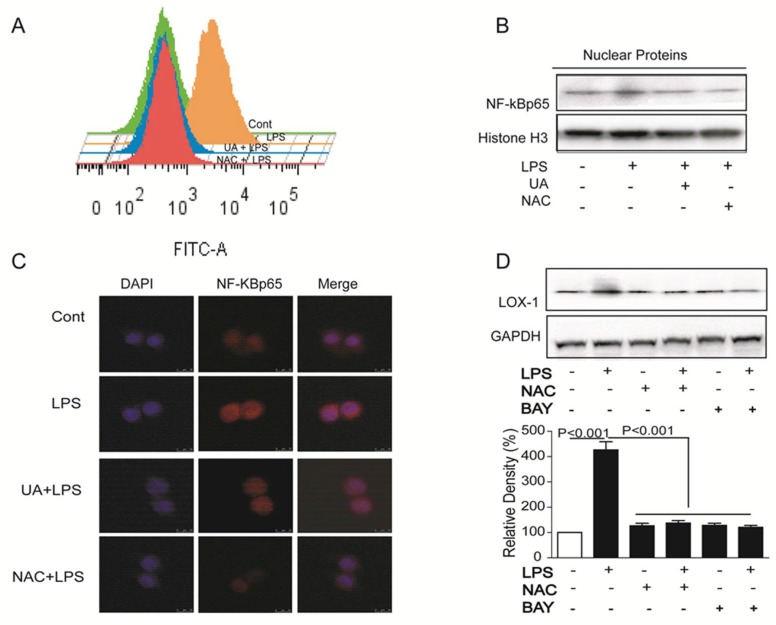
Cells were treated with LPS (5 mg/mL) for 4 h after pretreatment with UA (1 μM) or NAC (5 mM) for 1 h; ROS was determined by DCFH_2_-DA (**A**); Cells were treated with LPS (5 mg/mL) for 24 h with or without pretreatment with UA (1 μM), NAC (5 mM), or BAY (10 μM) for 1 h, and expression of p65 (**B**) and LOX-1 (**D**) was detected by western blotting; Cells were treated with LPS (5 mg/mL) with or without 1 h pretreatment with UA (1 μM) and NAC (5 mM), and p65 NF-κB localization was determined by immunofluorescence (**C**) (60×). UA, ursolic acid; BAY, BAY11-7082; NAC, N-acetyl cysteine.

ROS is one key signaling molecule involved in inflammation, and NF-κB pathway plays an essential role in LPS-induced LOX-1 expression in HUVECs [23]. In this study, LPS induced ROS production and NF-κB activity, while UA pretreatment obviously reduced ROS generation and blocked translocation of p65 NF-κB into nucleus (Figure 3A–C). Furthermore, all NAC (ROS scavenger) and BAY (NF-κB inhibitor) inhibited LOX-1 expression (Figure 3D), hinting that UA blocked ROS/ NF-κB pathway to regulate LOX-1 expression.

### 2.4. UA Reduced Atherosclerotic Plaque Development in ApoE^−/−^ Mice

**Figure 4:**
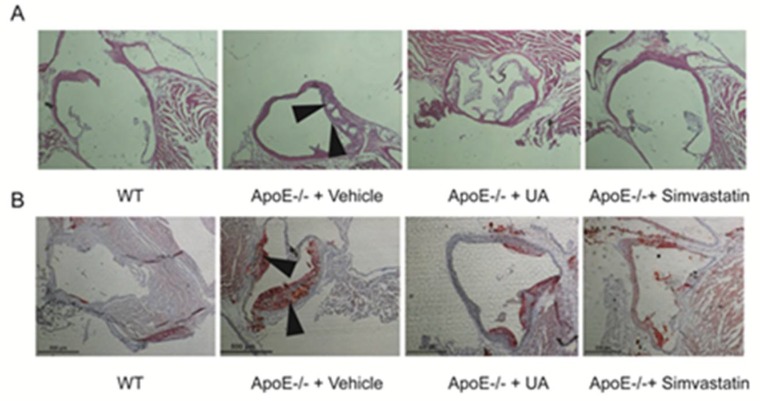
Aortic sinus sections were stained with Oil Red O and H&E to detect plaque sizes (40×) (**B**) and necrotic core areas (40×) (**A**), respectively. UA, ursolic acid; WT, wild type; ApoE^−/−^, apolipoprotein E-deficient.

Plaques formation and necrotic core areas occurrence are two key characteristics of atherosclerosis. In this study, both plaques and necrotic core areas were observed in artery of ApoE^−/−^ mice. UA administration significantly reduced plaque sizes as well as shrank necrotic core areas (Figure 4A,B).

### 2.5. UA Inhibited LOX-1 Expression in Thoracic Aorta of ApoE^−/−^ Mice

**Figure 5:**
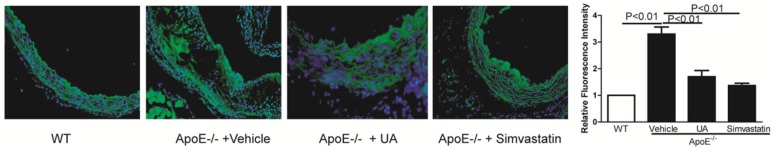
Expression of LOX-1 in the aorta was detected by immunofluorescence (4×). WT, wild type; ApoE^−/−^, apolipoprotein E-deficient; UA, ursolic acid.

LOX-1 was expressed at low level in aorta of WT mice but significantly increased in ApoE^−/−^ mice, which was dramatically decreased in UA pretreated mice (Figure 5).

## 3. Discussion

Due to the high life-threatening risk and severe financial burden of surgical treatment, pharmacological options for atherosclerosis therapy have always been discussed. UA, a natural pentacyclic triterpenoid carboxylic acid, is the major component of some traditional medicine herbs, although several studies have shown that UA has anti-inflammatory, anti-oxidative, and anti-diabetes functions [24,25]. There are conflicting studies around its anti-atherosclerosis effect. LOX-1 is the primary receptor for ox-LDL uptake, which promotes key steps involved in atherosclerosis [26,27,28,29]. In this study, LPS was chosen to induce endothelial LOX-1expression. UA decreased LOX-1 expression both in mRNA and protein levels, hinting that UA has potential anti-atherosclerosis activity.

Several lines of evidence support a role of oxidative stress in the pathogenesis of many diseases including atherosclerosis, tumor, Parkinson’s disease, and so on [30,31]. UA has been proved to own anti-oxidative effects. Experiment data showed that UA significantly reduced ROS generation, and ROS scavenger obviously decreased LOX-1 expression.

NF-κB plays a critical role in regulating inflammation and performs an important function in LPS-induced LOX-1 expression [23]. UA has been proved to own anti-inflammatory effects [32]. In this study, UA blocked p65NF-κB translocated into nucleus. Furthermore, NF-κB inhibitor decreased endothelial LOX-1 expression, suggesting the importance of NF-κB for regulating LOX-1.

The present findings in vitro allowed us to postulate that UA exhibited anti-atherosclerosis action in vivo. To verify this hypothesis, we detected the effects of UA on high-fat-diet-fed ApoE^−/−^ mice. The experiments showed that oral administration of UA for 12 weeks dramatically reduced plaque sizes and the shrinkage of necrotic core areas occurred in model ApoE^−/−^ mice providing the direct evidence for its anti-atherosclerotic effect. Besides, consistent with the in vitro results, expression of LOX-1 in the aorta was also decreased by UA, suggesting that LOX-1 is a promising therapeutic target in atherosclerosis for UA in future.

## 4. Materials and Methods

### 4.1. Materials and Reagents

UA (purity > 98%) was purchased from Chengdu Herbpurify Co. Ltd. (Chendu, China). Hoechst 33342, *N*-acetyl cysteine (NAC), LPS (Escherichia coli serotype 055:B5, LPS), 5-(6)-carboxy-2′, 7′-dichlorodihydrofluorescein diacetate (DCFH_2_-DA), and BAY11-7082 were purchased from Sigma Aldrich (St. Louis, MO, USA). Antibodies for TLR4, MyD88 were purchased from Santa Cruz Biotechnology (Santa Cruz, CA, USA). Antibodies for NF-κB p65, GAPDH, and Histone H3 were purchased from Cell Signaling Technology (Beverly, MA, USA). Anti-LOX-1 antibody was obtained from R&D (Minneapolis, MN, USA). The BCA protein kits were purchased from Thermo Fisher (Suwanee, GA, USA). SiRNAs for TLR4, MyD88 were purchased from Gene Pharma Company (Shanghai, China). Oil Red O Staining Kit and HE Staining Kit were obtained from Nanjing Jiancheng Bioengineering Research Institute (Nanjing, China).

### 4.2. Cell Culture

Human umbilical vein endothelial cells (HUVECs) (Gibco, Life Technologies Corp., Carlsbad, CA, USA) were cultured in Vascular Cell Basal Medium with Endothelial Cell Growth Kit-BBE at 37 °C in a humidified atmosphere of 5% CO_2_. Before passaging cells, issue culture flasks, 96-well plates, and 6-well plates were pre-coated with 0.1% gelatin. All assays were conducted using low cell passage cells (2–5 passages).

### 4.3. Animal Experiment

Male ApoE^−/−^ mice (6–8 weeks old) on C57BL/6J background and age-matched wild-type C57BL/6J controls were purchased from Beijing HFK Bioscience Co., Ltd. (Beijing, China). Mice were housed in SPF-grade animal facilities with a 12 h light/dark cycle at 23 °C (±2 °C). All animal procedures follow the NIH guide for the Care and Use of Laboratory Animals (NIH Publications No. 80-23, revised 1978). Starting from 6 weeks, the mice were fed with a HCD (54.35% raw grain, 20% lard, 0.15% cholesterol, 15% sucrose, 0.5% Sodium Cholate, and 10% yolk powder) for 12 weeks. All ApoE^−/−^ mice were dosed daily via intragastric gavage with 100 mg·kg^-1^·day^−1^ UA and 25 mg·kg^−1^·day^−1^ simvastatin dissolved in 0.5% CMC-Na or administered 0.5% CMC-Na alone (vehicle control) (*n* = 8 per group).

### 4.4. Immunofluorescence Assay

Cells (1 × 10^4^ cells/well) were seeded on glass slides in 96-well plates. After LPS treatment (with or without UA pretreatment), the slides were fixed with 4% PFA for 30 min. Then, the slides were permeabilized with PBS-T (containing 0.1% Triton x-100 in PBS solution) and blocked with PBS-B (containing 4% BSA in PBS solution). After being incubated with primary antibody (1:1000) and secondary antibody (1:5000), cells were stained with Hoechst 33342 in dark for 5 min. The protein location and expression were observed with IN Cell Analyzer 2000 (GE Healthcare).

### 4.5. Western Blotting

Treated HUVECs were washed twice with ice-cold PBS and lysed with RIPA buffer supplemented with a protease cocktail and phosphatase inhibitors. The cell lysates were separated using 8–10% SDS-PAGE and transferred onto PVDF membranes. After being blocked with 5% non-fat milk in TBST (20 mM Tris-HCl, 500 mM NaCl, and 0.1% Tween 20) at room temperature for 2 h, membranes were incubated with specific primary antibodies and secondary antibodies. The protein-antibody complexes were detected by ECL Advanced Western Blot Detection Kit. The intensity of the band was quantitated with Quantity One software (Bio-Rad).

### 4.6. SiRNA Transfection

Gene silencing experiment of TLR4, MyD88 with siRNA was performed according to our previous report [23]. Briefly, cells (1.0 × 10^6^/well) were seeded in 6-well plate overnight. 100 pM siRNA was diluted in 100 μL Opti-MEM reduced serum medium in each well and then mixed gently. Similarly, 5 μL of Lipofectamine TM 2000 was diluted in 100 μL of Opti-MEM reduced serum medium and mixed gently. After incubation for 5 min at room temperature, the diluted siRNA and diluted lipofectamine (total volume 200 μL) were mixed gently and incubated for another 20 min at room temperature. Further, siRNA-lipofectamine complex (200 μL) was added to each well. After incubation for 4 h, the complexes were discarded and cells were cultured with completed medium.

### 4.7. Real-Time RT-PCR

The mRNA expression of LOX-1 was determined with real-time PCR as our previous report [23]. Briefly, total RNA was extracted using TRIzol reagent (Life Technologies, USA). cDNA was synthesized by reverse transcription (RT) using GoScript™ Reverse Transcription System (Promega, Madison, WY, USA). Quantitative real-time PCR was carried out on Stratagene Mx3005P (Agilent Technologies, Santa Clara, CA, USA) using GoTaq^®^ qPCR Master Mix (Promega, USA). The primer sequences used in this experiment are listed for LOX-1: 5′-TGGGAAAAGAGCCAAGAGAA-3′ (forward), 5′- TAAGTGGGGCATCAAAGGA’ (reverse); anf for GAPDH: 5′-AGAAGGCTGGGGCTCATTTG-3′ (forward), 5′-AGGGGCCATCCACAGTCTTC-3′ (reverse). The levels of LOX-1 expression were determined by normalizing to GAPDH expression.

### 4.8. Aorta Collection and Lesion Size Evaluation

To evaluate plaque extension, frozen sections of the aortic sinus (8 mm) were stained using Oil Red O, hematoxylin, and eosin (H&E), respectively. Related experiments were performed following the method of Paigen et al. [33]*.*

### 4.9. Statistical Analysis

Twenty four ApoE^−/−^ mice were randomly allocated to groups and equal group sizes were obtained (*n* = 8 per group). Data were expressed as the means ± SD. The differences between groups were analyzed using Prism 5.0 (Graph Pad Software Inc., San Diego, CA, USA), and the statistical analysis was performed by analysis of variance (one-way ANOVA) followed by Student Newman–Keuls test.

## 5. Conclusions

In summary, this study showed that natural product-UA inhibited NF-κB-mediated LOX-1 expression both in vivo and in vitro through ROS generation. In view of the key roles of LOX-1 in the pathogenesis of atherosclerosis, inhibition of LOX-1 contributed to the anti-atherosclerotic effect of UA. Our study demonstrates that UA showed the effect of ameliorating atherosclerosis and put forward the potential mechanism. It supplies a new sight to illustrate the mechanism of action of UA for attenuates atherosclerosis in mice.

## Supplementary Materials

The Supplementary Materials are available online, Figure S1: the cytotoxic effect of UA on HUVECs.
