# Supplementary material for: Ursolic Acid Attenuates Atherosclerosis in ApoE−/− Mice: Role of LOX-1 Mediated by ROS/NF-κB Pathway
Source: Molecules. 2018 May 7;23(5):1101. doi: 10.3390/molecules23051101 (PMC6100321; doi:10.3390/molecules23051101)
Supplement: Supplementary file 1 [file molecules-23-01101-s001.pdf]

Supplementary Materials for

# Ursolic Acid Attenuates Atherosclerosis in ApoE-/- Mice: Role of LOX-1 Mediated by ROS/NF-κB Pathway

**Qiu Li [1], Wenwen Zhao [2], Xi Zeng [2], Zhihui Hao [3,*]**

[1] State Key Laboratory of Quality Research in Chinese Medicine, Institute of Chinese Medical Sciences, University of Macau, Macau SAR 999078, China; liqiu370725@126.com
[2] Department of Pharmacology, College of basic Medicine, Qingdao University, 308 Ningxia Road, Qingdao, Shandong, 266000, China; wenwenzhao0313@163.com (W.Z.); hf9079@163.com (X.Z.);
[3] Agricultural Bio-Pharmaceutical Laboratory, Qingdao Agricultural University, Qingdao, 266000, China; abplab@126.com (Z.H.)
* Correspondence: abplab@126.com; Tel.: +86-053288030364;

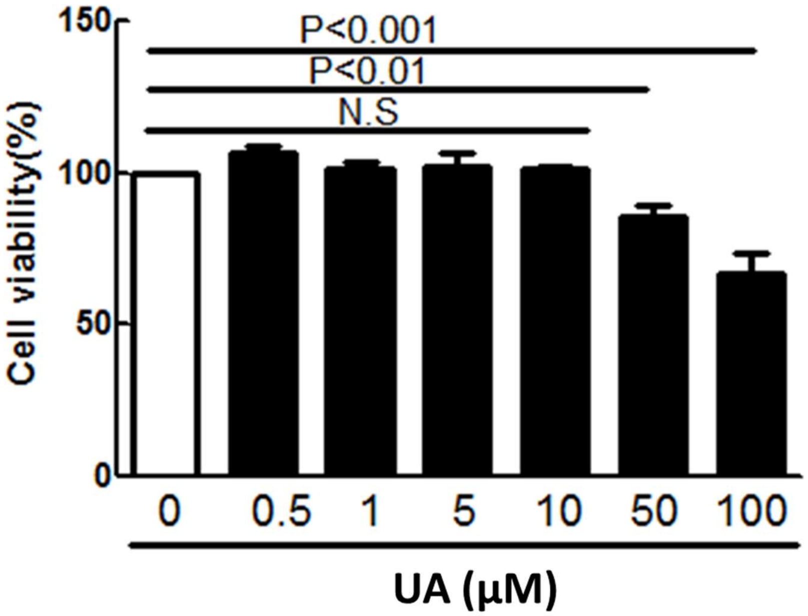


**Figure S1. The cytotoxic effect of UA on HUVECs**
